# Calycosin Alleviates Injury in Airway Epithelial Cells Caused by PM 2.5 Exposure via Activation of AMPK Signalling

**DOI:** 10.1155/2021/8885716

**Published:** 2021-05-04

**Authors:** Chunyan Wang, Jingjing Luo, Xiaoxue Bai, Shucheng Hua, Jing Jie, Han Liu, Jinying Gao, Lei Song

**Affiliations:** ^1^Cadre's Wards, the First Hospital of Jilin University, Changchun 130021, Jilin, China; ^2^Department of Respiratory Medicine, The First Hospital of Jilin University, Changchun 130021, Jilin, China

## Abstract

**Methods:**

Phospho-AMP-activated protein kinase (p-AMPK) and AMP-activated protein kinase (AMPK) were detected by western blot. Immunofluorescence staining was used to validate changes in the levels of nuclear factor kappa B (NF-*к*B) *p65* nuclear translocation. Mice were administered intraperitoneally with calycosin one hour before anaesthesia and endotracheal instillation of PM 2.5. The extent of lung injury was evaluated in the H&E-stained lung sections. Apoptotic cells were detected by TUNEL staining.

**Results:**

Administration of calycosin was increased in PM 2.5-treated B2B cells in a dose-dependent manner *in vitro*. Fluorescence signals from anti-NF-*к*B *p65* were increased in nuclei of cells pretreated with calycosin. The level of p-AMPK was increased by calycosin *in vitro* and *in vivo*. After pretreatment with compound C, the inhibitory effects of calycosin on cytotoxicity, levels of inflammatory cytokines and p-AMPK, and levels of NF-*к*B *p65* nuclear translocation were not significantly decreased *in vitro* or *in vivo*.

**Conclusions:**

Calycosin effectively decreased the release of inflammatory cytokines and alleviated injury caused by PM 2.5. These effects were mediated through activation of AMPK to suppress NF-*κ*B signalling.

## 1. Introduction

Long-term exposure to particulate matter ≤2.5 *μ*m in diameter (PM 2.5) is closely correlated with respiratory and cardiovascular diseases. Pun [1] found a positive association between 12-month moving average PM 2.5 exposure (per 10 *μ*g/m^3^ increase) and respiratory, chronic obstructive pulmonary diseases, and pneumonia mortality with risk ratios ranging from 1.10 to 1.24. The researchers also identified significant PM 2.5-associated risks for cardiovascular-related and lung cancer mortality in a cohort of 18.9 million Medicare beneficiaries living across the conterminous United States. PM 2.5 is a major component of air pollution in China that is increasingly affecting people's lives and health [2]. The annual average concentration of PM 2.5 is between 26 and 160 *μ*g/m^3^, and the average value is 72 *μ*g/m^3^, which is 2.06 times the annual average second-level standard (35 *μ*g/m^3^) of the environmental air quality standards in China [3]. The components of PM 2.5 largely determine its toxicity, including water-soluble inorganic ions, transition metals, and extractable organic matter (EOM) [4]. Given the complexity and variability of PM 2.5 formation, the characterisation of PM 2.5 toxicity and the identification of interventional measures will be of extreme practical significance [5].

Many Chinese herb extracts possess significant anti-inflammatory activities [6–8]. Calycosin, an isoflavonoid, is a major active component in Astragali radix that has been widely used to treat allergic inflammation in clinical practice [9]. This traditional herb has a long history of medicinal use in China and Southeast Asia. Calycosin also exerts antitumour and anti-inflammatory effects as an oestrogen receptor modulator (SERM) by inhibiting nuclear factor kappa B (NF-*κ*B) activation and mitogen-activated protein kinase (MAPK) phosphorylation [10–12]. Our previous studies showed that Let-7a can modulate PM 2.5-induced oxidative stress, and exposure to PM 2.5 induces aberrant activation of NF-*κ*B in human airway epithelial cells by downregulating microRNA-331 (miR-331) expression, revealing some pathological mechanisms of PM 2.5-induced injury of human airway epithelial cells [13, 14]. The present study investigated the effects of calycosin on the development of PM 2.5-induced injury in human airway epithelial cells, the possible mechanisms *in vitro* and *in vivo*. Due to its low cytotoxicity, calycosin could provide a novel natural medicine for alleviating PM 2.5-induced injury.

## 2. Materials and Methods

### 2.1. Collection of PM 2.5 Samples

PM 2.5 samples were collected from an urban area of Changchun, China, during November 2016 to March 2017, using 2500 QAT-UP Tissuquartz filters (Pall Life Sciences, Beijing, China). For purification, quartz filters containing PM 2.5 samples were cut into pieces, immersed in distilled water, and sonicated for 60 minutes using a water bath sonicator (Kun Shan Ultrasonic Instruments, Kunshan, China). The suspension was filtered through a sterile gauze, PM 2.5 samples were obtained by vacuum freeze-drying, and PM 2.5 was weighed.

### 2.2. Cell Culture

The Beas-2B (B2B) human bronchial epithelial cell line was purchased from the Cell Bank of the Chinese Academy of Sciences (Shanghai, China). B2B cells were cultured in Dulbecco's modified Eagle's medium (Gibco, Carlsbad, CA, USA) supplemented with 10% fetal bovine serum (Gibco), 100 U/mL penicillin and 100 mg/mL streptomycin. Cells were maintained in a humidified incubator at 37°C with 5% atmospheric CO_2_. The medium was refreshed every 3 days.

### 2.3. Calycosin Treatment

Calycosin was purchased from MCE Chemicals (Shanghai, China). The purity of calycosin is 99.89%. We used 1% DMSO to dissolve calycosin. In vitro experiments, various concentrations of calycosin were added to the wells. We added 20 *µ*L DMSO with 0 *µ*M, 1 *µ*M, 10 *µ*M, 50 *µ*M, 100 *µ*M, 500 *µ*M, 1000 *µ*M of calycosin. *In vivo* experiments, we added intraperitoneal injection of 25 mg calycosin per kilogram of mice weight, while the same volume of PBS was intraperitoneally administered to the negative control group.

### 2.4. PM 2.5 Exposure

*In vitro* experiments, a certain quantity of PM 2.5 was suspended and homogenised in DMEM supplemented with 2% FBS. We treated the B2B epithelial cell line with 100 *μ*g/mL concentration of PM 2.5 for positive groups and same dose of DMEM with 2% FBS for negative control group. *In vivo* experiments, we added 1 mg/mL PM 2.5 solution (100 *μ*L) into each mouse intranasal administration, while the same volume of PBS was intranasally administered to the negative control group.

### 2.5. Compound C Treatment

Compound C, an AMPK inhibitor, was purchased from Sigma-Aldrich (Shanghai, China). The purity of compound C is more than 98%. *In vitro* experiments, we used 1% DMSO to dissolve compound C to a concentration of 5 *µ*M, while the same volume of DMSO was administered to the negative control group. *In vivo* experiments, we administered tail vein injection of compound C (0.25 mg/kg), while the same volume of PBS intravenous injection was administered to the negative control group.

### 2.6. MTT Assay

B2B cells at a density of 2 × 10^4^ cells/mL were seeded in 96-well plates with 200 *µ*L in each well. After different treatments, cells were incubated in an incubator (37°C, 5% CO_2_) with 20 *µ*L of 3-(4,5-dimethylthiazol-2-yl)-2,5-diphenyltetrazolium bromide solution (MTT) (5 mg/mL) for 4 h. The medium containing MTT solution was removed, and 200 *µ*L of dimethyl sulphoxide was added. The spectrophotometric absorbance at 490 nm was determined using a microplate reader (Bio-Rad, PA, USA). Each experiment was performed in triplicate. Cell survival rate was then calculated using the equation: cell survival rate (%) = (values for the experimental group/values for the control group) × 100%

### 2.7. Quantitative Real-Time PCR (qRT-PCR)

Total RNA was exacted from B2B cells using TRIzol reagent (Invitrogen, Carlsbad, CA, USA), and cDNA was synthesised using EasyScript First-Strand cDNA Synthesis SuperMix (TransGen Biotech, Beijing, China) and used as a template. PCR was performed using TransStart SYBR Green qPCR SuperMix (TransGen Biotech) on a 7300 PCR system (ABI, Carlsbad, CA, USA). Commercial primers for IL-6 and IL-8, and the internal reference U6, were purchased from Guangzhou RiboBio (Guangzhou, China). Sequences of primers were 5′-GTAGCCGCCCCACACAGA-3′ (forward) and 5′-CATGTCTCCTTTCTCAGGGCTG-3′ (reverse) for IL-6 (101 bp); 5′-ATAAAGACATACTCCAAACCTTTCCAC-3′ (forward) and 5′-AAGCTTTACAATAATTTCTGTGTTGGC-3′ (reverse) for IL-8 (102 bp). Relative mRNA expression levels were calculated using the 2^−ΔΔCt^ method [13].

### 2.8. ELISA

Protein concentration was determined by the Bio-Rad protein assay reagent (Hercules, CA). Equal amounts (50 *μ*g) of proteins were loaded into respective enzyme-linked immunosorbent assay (ELISA) wells for assessment of IL-6 and IL-8 by using the kits obtained from BD Biosciences.

### 2.9. Western Blotting

Total protein from cells was extracted using RIPA buffer (Pierce, Rockford, IL, USA). The protein concentration was determined using the Bradford method (Pierce). Equal amounts of protein (40 *μ*g) were separated by 10% sodium dodecyl sulphate polyacrylamide gel electrophoresis (SDS_PAGE) and transferred to Immobilon-P membranes (Millipore, Bedford, MA, USA). After blocking with 5% nonfat milk in phosphate-buffered saline and Tween-20 for 1 h, membranes were incubated with anti-RAB14, anti-Akt, anti-p-Akt, anti-CCND1, anti-CDK2, or anti-Bax antibody (1 : 2000 dilution; Abcam, Hong Kong, China) as well as anti-GAPDH antibody (1 : 2000; Abcam) at 4°C overnight followed by incubation with horseradish peroxidase-conjugated secondary antibody (1 : 5,000 dilution) at 37°C for 1 h. Immunoreactive bands were detected using the ECL Plus Detection kit (Pierce).

### 2.10. Immunofluorescence Staining

B2B cells were fixed with 4% paraformaldehyde, washed, and incubated with rabbit anti-human NF-*к*B *p65* primary antibody (1 : 100 dilution; ABclonal, Woburn, MA, USA) overnight. Bound antibodies were detected with Alexa Fluor 647-conjugated donkey anti-rabbit IgG (1 : 200 dilution; Abcam, Cambridge, UK) and counterstained with 4′,6-diamidino-2-phenylindole (DAPI; Invitrogen). Fluorescence signals were captured using a fluorescence microscope (Olympus, Tokyo, Japan).

### 2.11. Mouse Model of PM 2.5 Instillation and Calycosin Treatment

Eight-week-old male C57/BL6 mice, with weight 20–25 g, were provided by the Animal Center of Jilin University and maintained in animal facility (20–25°C, 50–60% humidity, and 12-h light/12-h dark cycle with free access to sterilised food and water) in accordance with Chinese legislation on the use and care of laboratory animals. All procedures of animal experiments were approved by Animal Care and Use Committee of the First Hospital of Jilin University (Changchun, China). Mice were administered intraperitoneally with calycosin one hour before anaesthetised and endotracheal instillation of PM 2.5 for two weeks. All 48 C57/BL6 mice were randomly divided into four groups (*n* = 12): Group 1, negative control; Group 2, PM 2.5; Group 3, PM 2.5 + calycosin; Group 4, PM 2.5 + calycosin + compound C (through tail vein injection 30 mins before calycosin). The mice were sacrificed for further experiments.

### 2.12. Histological Analysis and Immunohistochemistry

The lung was inflated with 4% paraformaldehyde under 25 cm of water pressure and then embedded in paraffin. Paraffin blocks were sectioned to expose the maximum surface area of the lung tissue in the plane of the bronchial tree. Four micrometre sections were cut and stained with hematoxylin and eosin (H&E).

The extent of lung inflammation was evaluated in the H&E-stained lung sections. For lung injury scores, the characteristics of lung injury (alveolar capillary congestion, haemorrhage, inflammatory cell infiltration, alveolar wall thickness, and hyaline membrane formation) were analysed using the following criteria: 0, not present (normal); 1–4, 10–40% (mild); 5–6, 50–60% (moderate); 7–8, 70–80% (severe); 9–10, 90–100% (very severe) [15].

### 2.13. TUNEL Assay

The presence of apoptotic cells in lung tissue sections was determined using a terminal deoxynucleotide transferase dUTP nick end labelling (TUNEL) assay kit (Roche Diagnostics, Indianapolis, IN). The TUNEL assay (BD Biosciences) was carried out according to the manufacturer's protocol. TUNEL-positive cells are stained green. After the TUNEL assay, nuclei were stained with DAPI (blue fluorescence).

### 2.14. Statistical Analysis

All data are presented as the mean ± standard deviation (S.D.). Differences among different groups were analysed by one-way analysis of variance and post hoc Bonferroni tests using SPSS 23 (SPSS, Inc., Chicago, IL, USA) for Windows. A *p*-value <0.05 was considered statistically significant (^*∗*^). A *p*-value <0.01 was considered statistically significant (^*∗∗*^). A *p*-value <0.001 was considered statistically significant (^*∗∗∗*^).

## 3. Results

### 3.1. Calycosin Inhibits PM 2.5-Induced Cell Damage *In Vitro*

**Figure 1:**
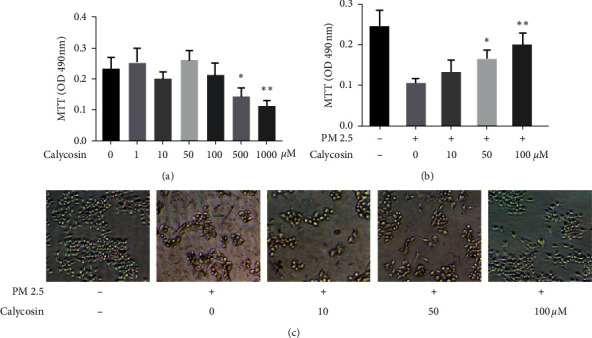
Calycosin inhibits PM 2.5-induced cell damage *in vitro*. (a) Absorbance of B2B cells at different calycosin concentrations. The P value between 0  *μ*m group and 500  *μ*m group for “^∗^” is 0.0356 < 0.05; the P value between 0  *μ*m group and 1000  *μ*m group for “^∗∗^” is 0.0097 < 0.01. (b) Absorbance of B2B cells treated with PM 2.5 at different calycosin concentrations. The P value between 0  *μ*m group and 50  *μ*m group for “^∗^” is 0.0180 < 0.05; the P value between 0  *μ*m group and 100  *μ*m group for “^∗∗^” is 0.0092 < 0.01. (c) Activity and number of B2B cells treated with or without PM 2.5 at different calycosin concentrations observed under a microscope at 40 × magnification.

The absorbance of B2B cells at 490 nm did not decrease significantly at calycosin concentrations of 1, 10, 50, or 100 *μ*M between 24 and 72 h (*p* > 0.05; Figure 1(a)) compared with the negative control group. However, the absorbance decreased significantly between the negative control group and the calycosin group of the concentration was 500 *μ*M and 1000 *μ*M (*p* < 0.05 and *p* < 0.01; Figure 1(a)). After exposure to PM 2.5, the absorbance of B2B cells decreased significantly than the negative control group (Figure 1(b)). The inhibitory effect of calycosin on PM 2.5-induced cell damage was evaluated at three concentrations (10, 50, and 100 *μ*M). Calycosin treatment significantly increased the absorbance of PM 2.5-treated B2B cells in a dose-dependent manner compared with cells not receiving calycosin. The differences were statistically significant when the calycosin concentration was 50 *μ*M and 100 *μ*M (*p* < 0.05 and *p* < 0.01, Figure 1(b)). The activity and number of B2B cells at 100 *μ*M calycosin were most similar to those of cells not exposed to PM 2.5 based on observation under a microscope at 40× magnification (Figure 1(c)).

### 3.2. Calycosin Inhibits PM 2.5-Induced Inflammation in B2B Cells

**Figure 2:**
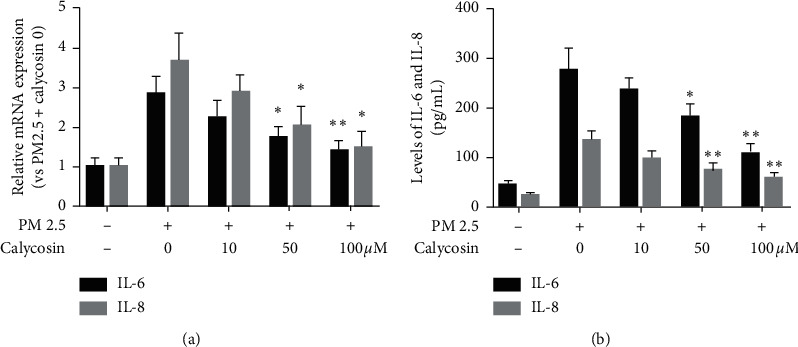
Calycosin inhibits PM 2.5-induced inflammation in B2B cells. (a) Relative levels of IL-6 and IL-8 mRNA transcripts. The P value between 0 um group and 50  *μ*m group for “^∗^” is 0.0216 < 0.05; the P value between 0  *μ*m group and 100  *μ*m group for “^∗∗^” is 0.0084 < 0.01 in IL-6. The P value between 0  *μ*m group and 50  *μ*m group for “^∗^” is 0.0328 < 0.05; the P value between 0  *μ*m group and 100  *μ*m group for “^∗^” is 0.0106 < 0.05 in IL-8. (b) Levels of IL-6 and IL-8 in the supernatants of cultured cells. The P value between 0  *μ*m group and 50  *μ*m group for “^∗^” is 0.033 < 0.05; the P value between 0  *μ*m group and 100  *μ*m group for “^∗∗^” is 0.0035 < 0.01 in IL-6. The P value between 0  *μ*m group and 50  *μ*m group for “^∗∗^” is 0.0099 < 0.01; the P value between 0  *μ*m group and 100  *μ*m group for “^∗∗^” is 0.0030 < 0.01 in IL-8.

PM 2.5 exposure upregulated proinflammatory IL-6 and IL-8 expression in B2B cells compared with the negative control group (Figure 2(a)). Calycosin at three concentrations (10, 50, and 100 *μ*M) inhibited the relative overexpression of IL-6 and IL-8 in cells pretreated with PM 2.5. However, the differences of IL-6 were statistically significant at concentrations of 50 *μ*M and 100 *μ*M (*p* < 0.05 and *p* < 0.05; Figure 2(a)); the differences of IL-8 were statistically significant at concentrations of 50 *μ*M and 100 *μ*M (*p* < 0.01 and *p* < 0.05; Figure 2(a)) compared with the PM 2.5 exposed group. Additionally, compared with the PM 2.5 exposed group, the levels of IL-6 and IL-8 were decreased significantly in the supernatant of cultured cells after treating with calycosin at 100 *μ*M (*p*-values were both less than 0.01; Figure 2(b)).

### 3.3. Calycosin Inhibits PM 2.5-Induced Activation of NF-кB Signalling in B2B Cells

**Figure 3:**
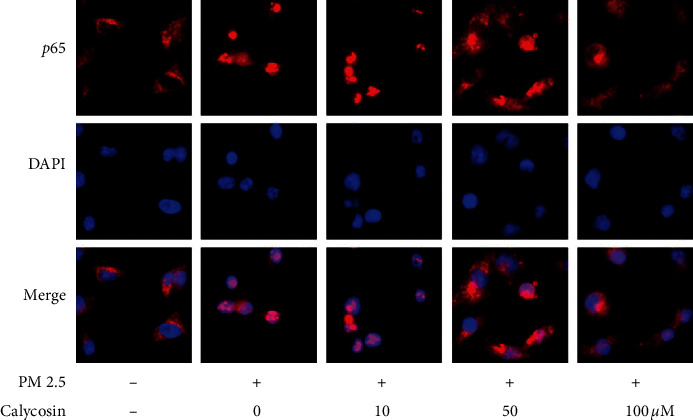
Immunofluorescence analysis of the NF-*κ*B *p65* distribution in B2B cells of different groups (40 × magnification; red = NF-*κ*B *p65*; blue = DAPI staining of nuclei).

Our previous study showed that exposure to PM 2.5 can activate NF-*к*B signalling by inducing NF-*к*B *p65* nuclear translocation [14]. To understand the molecular mechanisms by which calycosin inhibits the underlying toxicity of PM 2.5, B2B cells were exposed to PM 2.5 for 6 h, and changes in the levels of NF-*к*B *p65* nuclear translocation in different groups of cells (0, 10, 50, and 100 *μ*M calycosin) were determined by immunofluorescence. There was an obvious increase in the fluorescence signal from anti-NF-*к*B *p65* in the nuclei of cells at 6 h after exposure to PM 2.5. However, the fluorescence signal was not obviously increased in the nuclei of cells pretreated with calycosin (50 or 100 *μ*M) compared with untreated controls (Figure 3).

### 3.4. Calycosin Inhibits PM 2.5-Induced Cell Damage via the AMP-Activated Protein Kinase (AMPK) Pathway

**Figure 4:**
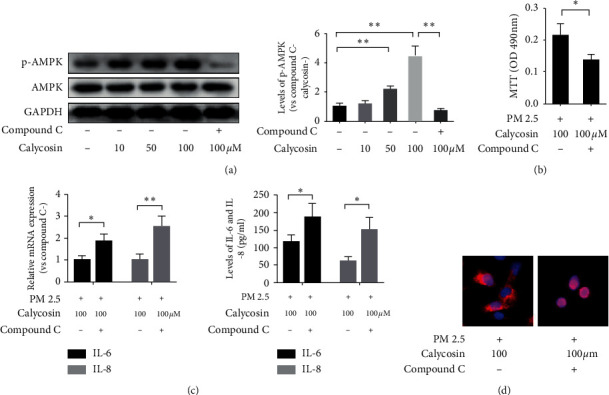
Calycosin inhibits PM 2.5-induced cell damage via the AMP-activated protein kinase pathway. (a) Levels of p-AMPK and AMPK in B2B cells. The P value between negative control group and 50  *μ*m group for “^∗∗^” is 0.0014 < 0.01; the P value between negative control group and 100  *μ*m group for “^∗∗^” is 0.0018 < 0.01; the P value between compound C group and 100  *μ*m without compound C group for “^∗∗^” is 0.0013 < 0.01. (b) The absorbance of B2B cells at 490 nm. The P value for “^∗^” is 0.0316 < 0.05. (c) Relative levels of IL-6 and IL-8 mRNA transcripts and levels of IL-6 and IL-8 in the supernatants of cultured cells. The P value for “^∗^” is 0.0175 < 0.05 in IL-6, and the P value for “^∗∗^” is 0.0086 < 0.01 in IL-8 for relative mRNA expression. The P value for “^∗^” is 0.0423 < 0.05 in IL-6, and the P value for “^∗^” is 0.0112 < 0.05 in IL-8 for the protein levels. (d) Immunofluorescence analysis of the NF-*κ*B *p65* distribution in B2B cells (400 × magnification; red = NF-*κ*B *p65*; blue = DAPI staining of nuclei).

Levels of phospho-AMPK (p-AMPK) were obviously increased following calycosin stimulation at 50 and 100 *μ*M compared with the negative control group (*p* < 0.01 and *p* < 0.01; Figure 4(a)). We also pretreated B2B cells with compound C, the specific inhibitor of AMPK, before calycosin (100 *μ*M) stimulation. The results showed that the level of p-AMPK in B2B cells pretreated with compound C was significantly lower than that in untreated cells after calycosin stimulation (*p* < 0.01; Figure 4(a)). The absorbance at 490 nm of B2B cells pretreated with compound C was also decreased significantly compared with the untreated group (*p* < 0.05, Figure 4(b)). Additionally, the relative mRNA expression in cells of IL-6 and the levels of IL-6 in the supernatant pretreated with compound C were elevated compared with those in the untreated group (*p* < 0.05 and *p* < 0.05; Figure 4(c)), and IL-8 in cells and the supernatant were increased compared with those in the untreated group (*p* < 0.01 and *p* < 0.05; Figure 4(c)). Furthermore, the fluorescence signal from anti-NF-*к*B *p65* was obviously increased in the nuclei of cells after treatment with compound C (Figure 4(d)).

### 3.5. Calycosin Inhibits PM 2.5-Induced Inflammation in Animals via the AMP-Activated Protein Kinase (AMPK) Pathway

**Figure 5:**
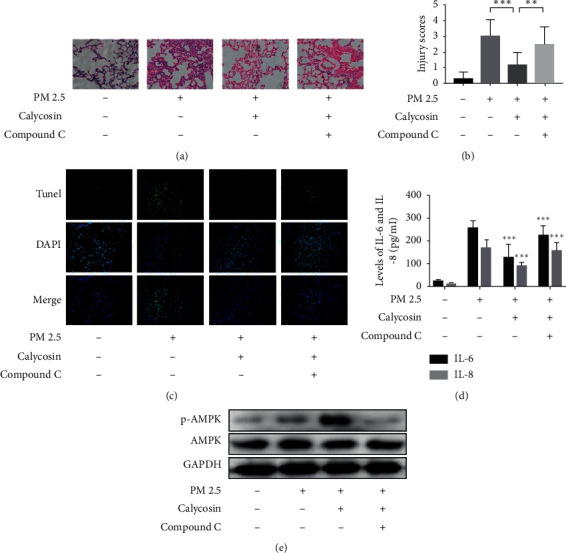
Calycosin inhibits PM 2.5-induced inflammation *in vivo*. (a) Lung tissue with infiltration of inflammatory cells around the bronchus and vessel after instillation of PM 2.5. (b) Lung injury scores in lung tissue for PM 2.5-instilled mice. (c) Apoptosis was measured by TUNEL assay. (d) The levels in supernatant of IL-6 and IL-8 in mice. (e) Levels of p-AMPK and AMPK *in vivo*.

Examples of the lung tissue with infiltration of inflammatory cells around the bronchus and vessels after instillation of PM 2.5 are shown in Figure 5(a). An average score of 3 for lung injury in the PM 2.5-instilled group were shown in Figure 5(b). Treatment of calycosin could significantly decrease the lung injury compared with the intranasally administered PM 2.5 group (*p* < 0.001; Figure 5(b)). While compound C enhanced lung injury compared with the calycosin treatment group (*p* < 0.01; Figure 5(b)), there was no difference in the PM 2.5 group (*p* > 0.05; Figure 5(b)). The TUNEL staining demonstrated that TUNEL-positive cells were observed in the PM 2.5 groups, but those in the calycosin treatment group were markedly decreased (Figure 5(c)). Calycosin had the strongest inhibitory effect on IL-6 and IL-8 overexpression induced by PM 2.5 exposure compared with the negative control group (*p* < 0.001 and *p* < 0.001; Figure 5(d)). Additionally, levels of IL-6 and IL-8 increased significantly after treatment of calycosin and compound C compared with the calycosin group (*p* < 0.001 and *p* < 0.001; Figure 5(d)). Levels of phospho-AMPK (p-AMPK) were obviously increased following calycosin stimulation (Figure 5(e)). The results showed that the level of p-AMPK pretreated with compound C was significantly lower than that in calycosin-treated group.

## 4. Discussion

Our previous findings demonstrated that PM 2.5 exposure induces reactive oxygen species (ROS) production, leading to inflammation and damage in various types of cells [13, 16–18]. In the present study, we found that calycosin significantly alleviated cell damage and reduced the levels of IL-6 and IL-8 induced by PM 2.5 exposure at a concentration of 100 *μ*M, with low cytotoxicity at the same concentration. These results suggested that calycosin markedly reduced PM 2.5-induced oxidative stress and cell injury. In addition, *in vivo*, our study also proved that calycosin can reduce the acute lung injury caused by PM 2.5 via the AMP-activated protein kinase (AMPK) pathway. Liu [19] reported a recipe of several herbal medicines that reduced PM 2.5-induced lung injury in rats. To the best of our knowledge, the present work is the first to report a reduction in PM 2.5-induced damage *in vitro* and *in vivo* by a single Chinese herb extract.

Our previous study supported the notion that PM 2.5 exposure induces aberrant activation of NF-*к*B signalling in human airway epithelial cells [15, 18, 20, 21]. Active NF-*к*B can be translocated from the cytosol to the nucleus, where it activates the transcription of various genes, including IL-6, tumour necrosis factor-*α* (TNF-*α*), interluekin-1*β* (IL-1*β*), and inducible nitric oxide synthase (iNO_S_), which are involved in the inflammatory response and lead to cell injury [22]. Tao et al. [9] found that calycosin may also exert effects by inhibiting the activation of the Toll-like receptor-4 (TLR4)-mediated NF-*к*B signalling pathway. Thus, we explored the effects of calycosin on PM 2.5-induced activation of NF-*к*B signalling. The results showed that calycosin inhibited the activation of NF-*к*B signalling induced by PM 2.5 exposure in B2B cells. Because NF-*к*B signalling is crucial for the regulation of oxidative stress and inflammatory responses [23], we inferred that calycosin relieves PM 2.5-induced oxidative stress via NF-*к*B signalling, which contributes to the pharmacological mechanism of calycosin in alleviating PM 2.5 exposure-induced cellular injury.

Accumulating evidence indicates that the regulation of innate immunity and energy metabolism are connected through antagonistic crosstalk [24]. Recent studies reported that medicine-activated AMPK may participate in modulating the expression of inflammatory cytokines through NF-*к*B [22, 25–27]. AMP-activated protein kinase (AMPK), an upstream protein of NF-*к*B, is a critical signalling macromolecule and key cellular metabolic sensor for maintaining the ADP/AMP/ATP levels [28]. As an energy sensor, AMPK regulates cellular metabolism and homeostasis and promotes autophagy [29]. There is increasing evidence that, in many cell types, an increase in intracellular ROS can activate p-AMPK [30]. Herein, p-AMPK was increased following calycosin treatment. After pretreated with compound C, the inhibitory effect of calycosin on PM 2.5-induced inflammatory responses and cytotoxicity were decreased significantly, accompanied by lower p-AMPK levels *in vitro* and *in vivo*. These findings indicated that calycosin-activated AMPK signalling in human airway epithelial cells and in mice may inhibit NF-*к*B, providing new insights into the pharmacological mechanism of calycosin in the prevention and treatment of PM 2.5-induced injury of human epithelial cells. Therefore, strategies using calycosin to activate AMPK signalling may provide alternatives to current clinical approaches for inhibiting inflammatory responses and preventing cell damage. However, the exact mechanisms of calycosin in PM 2.5-induced damage *in vitro* and *in vivo* need to be further investigated using more specific experimental approaches.

## 5. Conclusions

In summary, calycosin, a traditional Chinese herb extract, can effectively decrease the release of inflammatory cytokines and alleviate injury in PM 2.5-induced airway epithelial cells *in vitro* and *in vivo*. These effects of calycosin were related to the activation of AMPK to suppress NF-*κ*B signalling. Our results suggested that calycosin should be considered a potentially potent anti-inflammatory candidate for the treatment or prevention of PM 2.5-induced cell damage.

## Data Availability

The data sets used and/or analysed during the current study are available from the corresponding author on reasonable request.

## Abbreviations

PM 2.5: Particulate matter ≤2.5 *μ*m
MTT assay: 3-(4,5-Dimethylthiazol-2-yl)-2,5-diphenyltetrazolium bromide assay
B2B: Beas-2B
H&E: Hematoxylin and eosin
IL-6: Interleukin-6
IL-8: Interleukin-8
p-AMPK: Phospho-AMP-activated protein kinase
AMPK: AMP-activated protein kinase
NF-*κ*B: Nuclear factor kappa B
SERMs: Oestrogen receptor modulators
MAPKs: Mitogen-activated protein kinases
qRT-PCR: Quantitative real-time polymerase chain reaction
DAPI: 4′,6-Diamidino-2-phenylindole
TUNEL: Transferase dUTP nick end labelling
ROS: Reactive oxygen species
TNF-*α*: Tumour necrosis factor-*α*
IL-1: Interleukin-1*β*
iNO_S:_: Inducible nitric oxide synthase
TLR-4: Toll-like receptor-4.
